# Polarization independent dielectric metasurface for infrared beam steering applications

**DOI:** 10.1038/s41598-019-47097-5

**Published:** 2019-07-25

**Authors:** Mostafa Abdelsalam, Ahmed M. Mahmoud, Mohamed A. Swillam

**Affiliations:** 1 Department of Physics, School of Sciences and Engineering, The American University in Cairo, Cairo, 11835 Egypt; 2 Electronics and Communications Engineering Department, The American University in Cairo, Cairo, 11835 Egypt

**Keywords:** Metamaterials, Electrical and electronic engineering

## Abstract

Over the past years, metasurfaces have been of great interest due to their ability manipulate optical wavefront by introducing a phase gradient across the transverse directions of the wave. This phase gradient was usually realized using plasmonic resonators which had high intrinsic losses. Here, we demonstrate, numerically, a proof of principle of an all-dielectric silicon based metasurface at the infrared (IR) range that manipulates the wave front and achieves beam steering with significantly high transmission. The proposed cross-shaped unit cell design shows high transmission with the ability to fully control the phase of the transmitted wave from 0 to 2π. The metasurface is made of silicon cross resonators, arranged to have a linear phase gradient, on SiO_2_ substrate which makes the device compatible with most standard semiconductor fabrication techniques.

## Introduction

Metasurfaces are planar (or semi-planar) structures based on a periodic or quasi periodic two-dimensional array of cells. Arranging these cells in a certain manner can be used to manipulate certain properties of light^1–4^. Conventional devices controlled the light wave by accumulating phase through propagating through a certain medium. In metasurfaces however, each unit cell conveys a discrete phase change for the incident light^1^. Through the proper design of the unit cells we can control different properties of the light wave such as amplitude, phase and polarization. Many structures have been proposed to demonstrate wide range of applications for metasurfaces, such as lenses^5,6^, focusing mirrors^7^, and holograms^8^. Metasurfaces were engineered based on metal planar structures, which have significantly high intrinsic losses that lead associated low transmission limiting their practical applications^1,6,7,9–11^. On the other hand, all-dielectric resonant metasurfaces have negligible absorption losses^12,13^. For most of the proposed designs, all-dielectric metasurfaces demonstrate superior performances in terms of transmission and control over both polarization and phase gradient across the interface. These structures are mostly operating at visible, near-IR or telecom frequencies^7,14–18^. However, applications of metasurfaces at longer IR wavelengths have a lot of potential^19,20^. The IR is range of great interest for a variety of applications, as the thermal signatures of most objects exist in this range. Hence, it can be used for sensing applications. In addition, it is also essential for thermal imaging and energy harvesting^21^. There have been numerous efforts for designing IR beam-steering metasurfaces^16,22^. However, it is still challenging to handle two different polarizations, that are normal to each other, simultaneously to achieve polarization independence. In this work, a polarization-independent unit cell is developed and utilized within an all-dielectric metasurfaces that is CMOS compatible. This new design can achieve beam steering with normalized transmission as high as 0.89 for x-polarized field and 0.81 for y-polarized light. This design can be tailored to achieve different steering angles by simply changing the periodicity of the structure.

## Methods and Results

**Figure 1:**
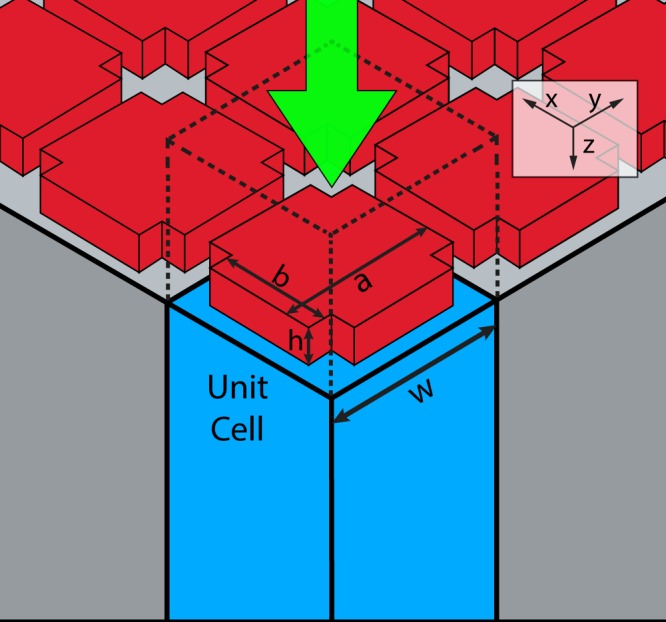
Schematic of the proposed periodic structure where the enclosed area represents a single unit cell.

To achieve polarization independence, the unit cell must be symmetric in the transverse directions. Additionally, this unit cell should exhibit both electric and magnetic resonances in the vicinity of each other around the operation wavelength (λ_o_). This would allow us to fully control the phase of the transmitted wave over the range from 0 ° to 360^o^. A cross-shaped unit cell is chosen to achieve the previously mentioned design constraints (Fig. 1). Choosing the design parameters to be the length (a) and width (b) of the cross will maintain the symmetry in the transverse direction. This cross is made of silicon with refractive index of 3.67 on a SiO_2_ substrate of refractive index 1.45^22^. Height (h) of 560 nm was found to be sufficient to manipulate both electric and magnetic resonances for the operation wavelength (*λ*_*o*_) of 3.1 µm. This is achieved using periodicity (w) of 1.6 µm. This satisfies the condition of the unit cell being less than half the operation wavelength.

**Figure 2:**
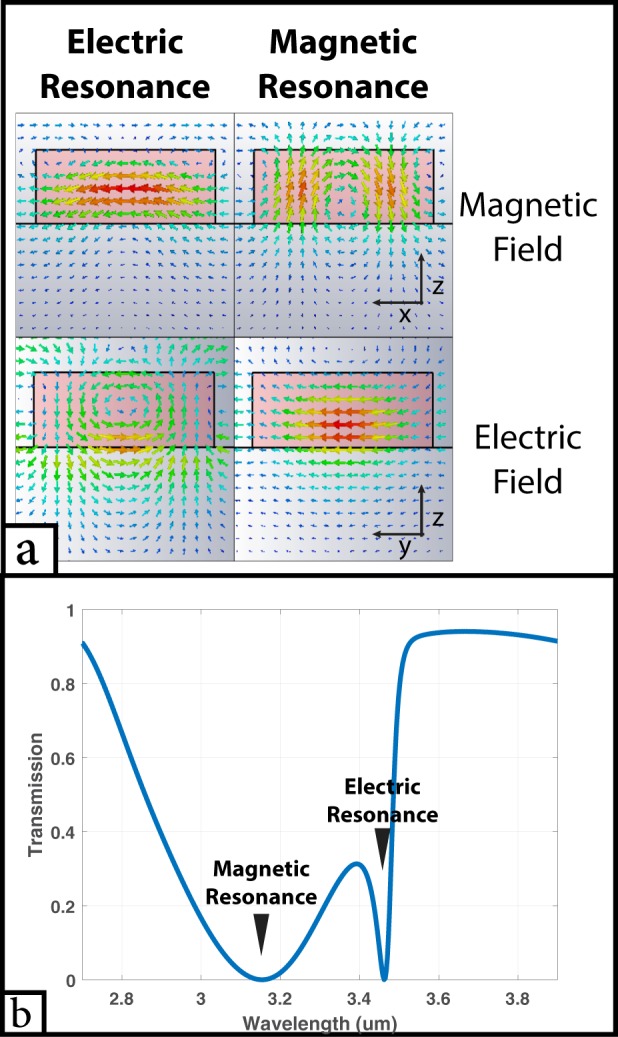
(**a**) Electric and magnetic fields at both electric and magnetic resonance wavelengths when a = 1.3 µm, and b = 0.9 µm. (**b**) Transmission spectrum at the same dimensions.

Finite difference time domain (FDTD) simulations have been used to study the behavior of the previously mentioned structure. A unit cell having of a = 1.3 µm and b = 0.9 µm was used to check the resonance effect discussed previously. A y-polarized plane wave is injected in the z-direction and the transmission spectrum is studied. Two resonance peaks can be seen in Fig. 2b, one at 3.46 µm and the other at 3.18 µm. At 3.46 µm, the electric field in the y-z plane resembles that of a dipole and the magnetic field in the x-z plane has a vortex like shape and vice versa at 3.18 µm (Fig. 2a). This verifies that the resonance at 3.46 µm is indeed electric resonance while at 3.18 µm is magnetic resonance.

**Figure 3:**
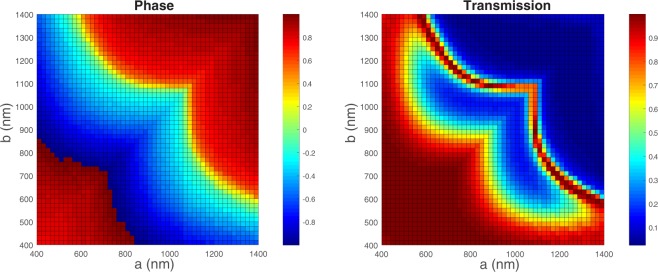
Phase (in degrees) and normalized transmission chart for different a and b at the operation wavelength which is 3.1 μm.

Next, a sweep over the design parameters a and b has been performed to find the phase and transmission corresponding to each dimension of a and b. This gives us a clear map for the required dimensions to achieve a specific transmission and its corresponding phase (Fig. 3). This can be used in different applications like beam steering or lensing.

**Figure 4:**
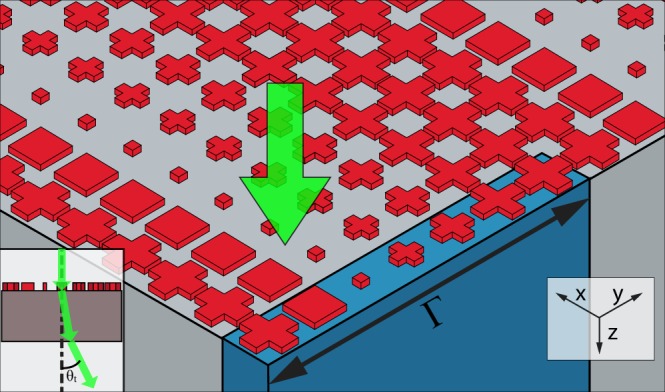
3D schematic for the steering structure. The inset is a side view of the structure.

To achieve beam steering, we can divide the entire phase range into seven segments having 51° increment to the phase. In our case we approximated this to 50° increment (i.e. 0, 50, 100, 150, 200, 250, 300) with a = 1.38, 1.08, 0.28, 0.8, 1.04, 1.34, 1.4 µm and b = 0.56, 0.97, 0.28, 0.3, 0.38, 0.42, 0.5 µm. This creates a phase gradient along one of the transverse directions (in our case we chose this to be the y-direction). This gradient introduces an extra term to Snell’s law as follows^1^:

1 $${n}_{s}\,\sin ({\theta }_{s})-{n}_{i}\,\sin ({\theta }_{i})=\frac{{\lambda }_{o}}{2\pi }\frac{d{\rm{\Phi }}}{dx}$$

where n_i_ and n_s_ are the refractive indices in the background material and substrate respectively. θ_s_ and θ_i_ are the angles of transmission and incidence, and $\frac{d{\rm{\Phi }}}{dx}$ is the phase gradient along x. In our case n_i_ is 1 for air and n_s_ is 1.45 for SiO_2_. Using this equation at the first interface and the normal Snell’s law at the other interface between the substrate and air we can deduce that for a ray incident normally on the metasurface then propagating through the substrate to air again the angle of transmission will follow the equation^22^:

2 $${\theta }_{t}={\sin }^{-1}(\frac{{n}_{i}Sin{\theta }_{i}+\frac{{\lambda }_{o}}{{\rm{\Gamma }}}}{{n}_{t}})$$

where θ_t_ is the transmission angle, n_t_ is the refractive index of air, and Γ is the periodicity of the structure in the direction where there is a phase gradient, which is the y-direction in our case (Fig. 4). From this we can expect θ_t_ to be about 16°.

**Figure 5:**
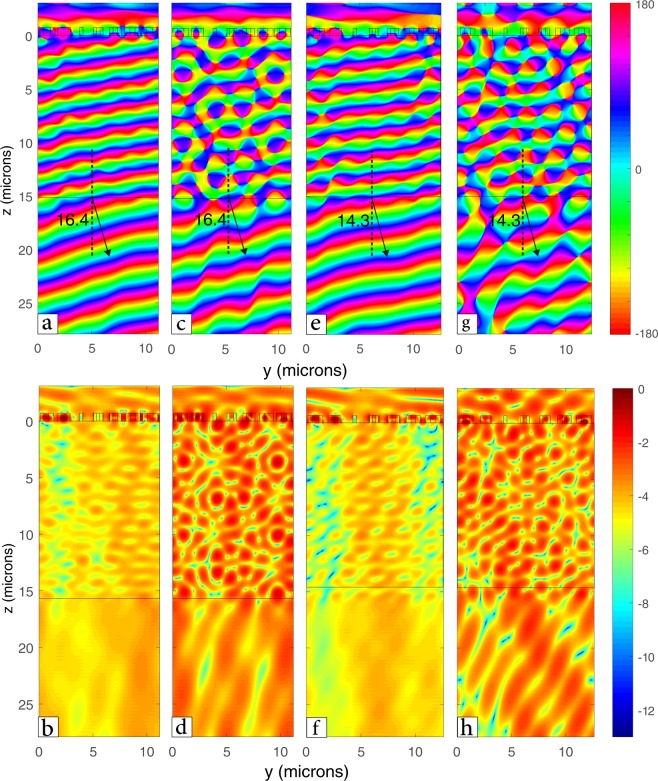
Field propagation after passing by the beam steering structure showing (**a**) the phase of a y-polarized plane wave with w = 1.6 µm, (**b**) log scale of the normalized intensity of a y-polarized with w = 1.6 µm, (**c**) the phase of an x-polarized plane wave with w = 1.6 µm, (**d**) log scale of the normalized intensity of an x-polarized with w = 1.6 µm, (**e**) the phase of a y-polarized plane wave with w = 1.8 µm, (**f**) log scale of the normalized intensity of a y-polarized with w = 1.8 µm, (**g**) the phase of an x-polarized plane wave with w = 1.8 µm, and (**h**) log scale of the normalized intensity of an x-polarized with w = 1.8 µm at the operation wavelength of 3.1 µm.

A plane wave is injected on the metasurface in the z-direction. Both polarizations have been simulated to ensure polarization independence. Note that although the unit cell is symmetric, the beam steering structure itself is not symmetric in x and y. It is expected to see small difference between the x and y-polarized fields (Fig. 5a–d). In the case of y-polarization the normalized transmission was found to be 0.8 and the wave fronts were more regular than that found in the x-polarized field. However, when using y-polarized field the transmission is 0.89 which is the greatest beam steering efficiency achieved to date according to our knowledge.

The beam steering angle can be changed by simply manipulating Γ. This is done by increasing the unit cell dimension to Γ = 12.6 µm. This successfully changed the angle to 14.3° which is expected from Eq. (2) (Fig. 5e–h). However, the transmission was reduced to 0.53 this was due to the change of the periodicity of each cross from the initial design (i.e. from 1.6 to 1.8 µm).

**Figure 6:**
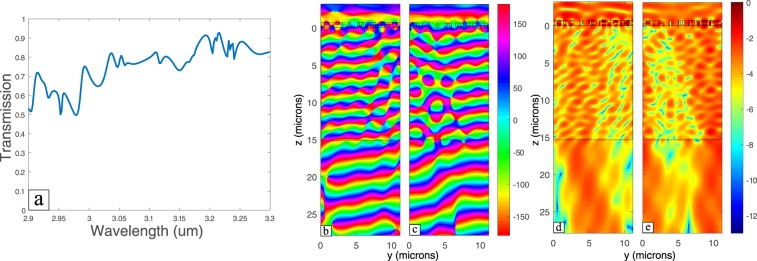
(**a**) Transmission spectrum for the steering structure. (**b**) Field propagation of y-polarized plane wave at a wavelength of 2.96 µm (**c**) and at a wavelength 3.2 µm.

The operation bandwidth is defined as the range of wavelengths where beam steering effect still occur with change of the transmission ±10% and the angle doesn’t deviate ±5° from the design steering angle that can obtained for each wavelength from Eq. (2). It was found that the device can have an operation bandwidth ranging from 2.96 µm to 3.2 µm. At 2.96 µm the steering angle is 12° which is less than the design angle for this wavelength found from Eq. (2) only by 3.32° (Fig. 6b). At 3.2 µm the steering angle is 17.5° which is more than the design angle for this wavelength found from Eq. (2) only by 0.9° (Fig. 6c). Although the transmission is still relatively high outside the operation bandwidth (Fig. 6a), the wave front starts to distort and the steering function is compromised.

**Figure 7:**
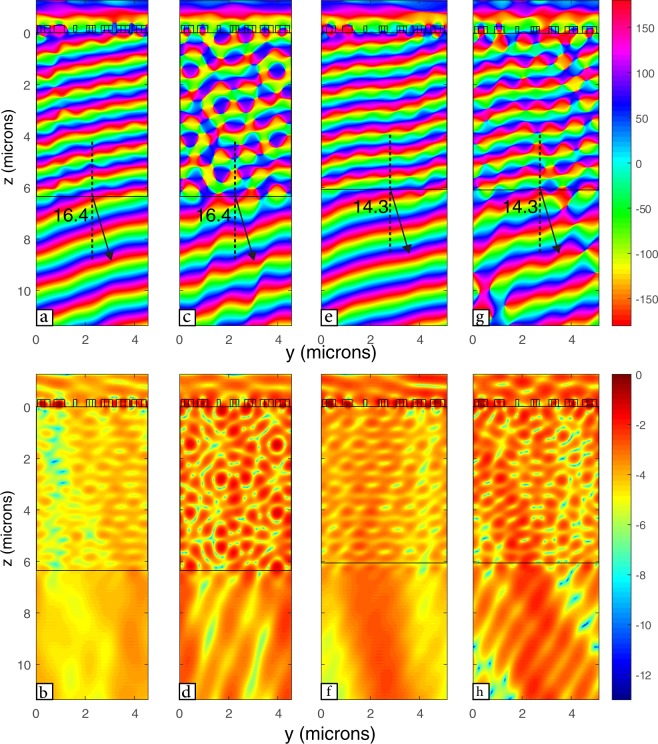
Field propagation through structure designed with thickness 220 nm for operation wavelength 1.218 µm showing (**a**) the phase of a y-polarized plane wave with w = 0.65 µm, (**b**) log scale of the normalized intensity of a y-polarized with w = 0.65 µm, (**c**) the phase of an x-polarized plane wave with w = 0.65 µm, (**d**) log scale of the normalized intensity of an x-polarized with w = 0.65 µm, (**e**) the phase of a y-polarized plane wave with w = 0.733 µm, (**f**) log scale of the normalized intensity of a y-polarized with w = 0.733 µm, (**g**) the phase of an x-polarized plane wave with w = 0.733 µm, and (**h**) log scale of the normalized intensity of an x-polarized with w = 0.733 µm.

We also show that this structure can support scalability. We scale the structure to have a smaller height (h = 220 nm) so the structure can be fabricated using standard Silicon-on-Insulator (SOI) fabrication procedure. This required that the dimensions of the whole structure be multiplied by 0.393, which is the ratio between the old and new heights. The structure works in the near-infrared domain with operation wavelength of 1.218 µm. Figure 7(a–d) shows that similar beam steering effect has been simulated for different x and y-polarized plane waves, with steering angle of 16.4°. While changing the periodicity (w) to be 0.733 µm instead of 0.65 µm decreases the steering angle to 14.3° (Fig. 7e–h). This scaling of the height shows the flexibility of the design to different wavelengths.

## Conclusion

A new polarization-independent unit cell design was developed for all-dielectric metasurfaces that can be CMOS compatible. This new design can achieve beam steering with the highest efficiency to date of 0.89 for x-polarized field and 0.81 for y-polarized light. This design can be tailored to achieve different steering angles by changing the periodicity of the structure. The operation bandwidth was studied and found to be narrow from 2.96 µm to 3.215 µm.

## Data Availability

All data needed to evaluate the conclusions in the paper are present in the paper.
